# Risk of coronary heart disease in patients with periodontitis among the middled-aged and elderly in China: a cohort study

**DOI:** 10.1186/s12903-021-01951-z

**Published:** 2021-12-07

**Authors:** Kaikai Gao, Zhiyuan Wu, Yue Liu, Lixin Tao, Yanxia Luo, Xinghua Yang, Jingbo Zhang, Xiuhua Guo, Songlin Wang

**Affiliations:** 1 School of Public Health, Capital Medical University, No.10 Xitoutiao, You’anmen Wai, Fengtai District, Beijing, 100069 China; 2 Beijing Laboratory of Oral Health, Capital Medical University, No.10 Xitoutiao, You’anmen Wai, Fengtai District, Beijing, 100069 China; 3 National Institute for Data Science in Health and Medicine, Capital Medical University, Beijing, China; 4 Department of Epidemiology and Health Statistics, School of Public Health, Capital Medical University, Beijing, China; 5 Beijing Physical Examination Center, Beijing, China; 6 Beijing Municipal Key Laboratory of Clinical Epidemiology, Beijing, China; 7 Department of Biochemistry and Molecular Biology, Capital Medical University School of Basic Medical Sciences, Beijing, People’s Republic of China

**Keywords:** Periodontitis, Coronary heart disease, Oral health, Cohort study

## Abstract

**Background:**

Convincing evidence of the periodontitis as a risk factor for coronary heart disease (CHD) is lacking due to shared risk factors, and no cohort study has investigated the association between CHD and periodontitis in Chinese populations.

**Methods:**

This study used a prospective cohort study design. The analysis included 4591 participants aged 40 years and older (3146 men and 1445 women). The association between CHD and periodontitis was estimated using relative risk (RR) calculated using modified Poisson regression. Multiple mediation analysis was used to differentiate the relative effects (RE) from different risk factors on the effect of periodontitis on CHD.

**Results:**

In the analysis using the imputed dataset and fully adjusted model, participants with periodontitis at baseline had 37% increased risk of CHD overall compared to those without periodontitis at baseline (RR 1.37; 95% CI 0.96–1.95). Most of the association can be explained by age, sex, history of diabetes, history of hypertension, uric acid and education (RE 0.76; 95% CI 0.41–1.02).

**Conclusion:**

Periodontitis was weakly associated with an increased risk of CHD among the middled-aged and elderly in China. Further studies are required to identify more mediators and elucidate the mechanisms of how periodontitis increases the risk of CHD.

**Supplementary Information:**

The online version contains supplementary material available at 10.1186/s12903-021-01951-z.

## Background

Periodontitis is an inflammatory disease that affect the supporting structures of the teeth, which could lead to tooth loss and contribute to systemic inflammation [1]. Bacteremia and systemic inflammatory caused by periodontitis are important factors in the initiation of the endothelial lesion as well as in the potentiation of the vascular wall inflammatory process that lead to the development of atherosclerosis causally [2]. Chronic infections due to periodontitis is one of the most common chronic infections have been implicated in the pathogenesis of atherosclerosis [3].

Although periodontitis as a risk factor for CHD is plausible biologically, convincing evidence is lacking [4–6]. It is difficult to interpret the association due to common risk factors such as diabetes and smoking are shared between CHD and periodontitis [7, 8].

According to the China’s Fourth National Oral Health Epidemiological Survey of 2017 [9], periodontal health condition becomes increasingly worse among the middled-aged and elderly in China. Meanwhile, CHD is the second leading cause of cardiovascular death in the Chinese population [10]. Unfortunately, there were no cohort studies estimating the association between CHD and periodontitis in Chinese populations. We aimed to speculate whether periodontitis is a direct risk factor for CHD among the middled-aged and elderly in China and quantify mediation/confounding effects due to shared factors.

## Methods

### Study design and participants

**Fig. 1:**
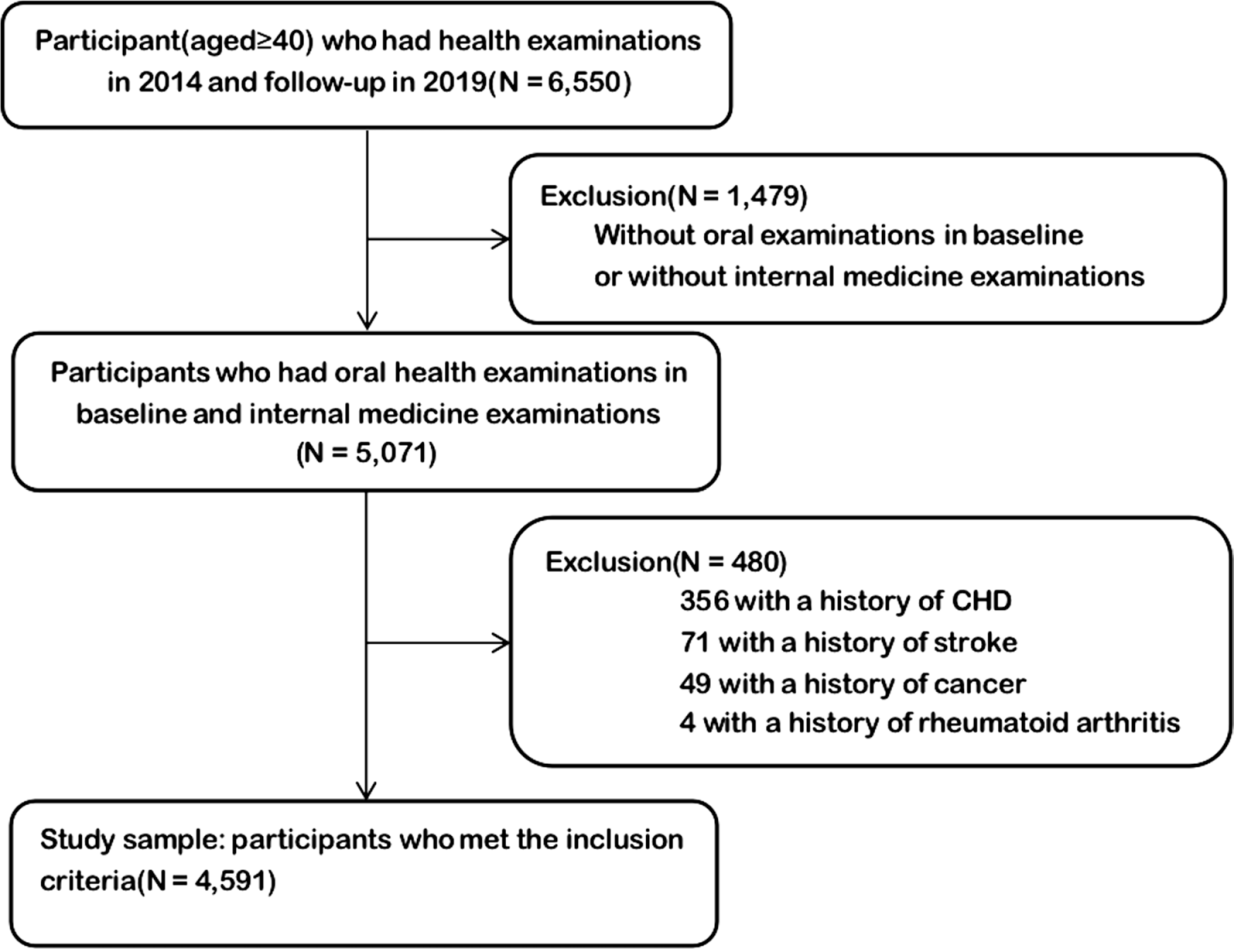
Flow chart of the study population

The Beijing health management cohort (BHMC) is a large prospective dynamic cohort study established in 2008 in Beijing, China. The BHMC study was conducted based on health examination populations from the Beijing Xiaotangshan Examination Center and Beijing Physical Examination Center. The recruited participants were asked to take an annual health examination, including physical examination (height, weight, blood pressures), face-to-face questionnaire survey (demographic variables, lifestyles, diseases history) and biochemical examination. BHMC was designed to investigate the risk factors and biomarkers for metabolism-related diseases. Details of the study design have been described previously [11]. In this study, we used a prospective cohort study design. This longitudinal cohort consisted of 6550 participants aged 40 years and older attended health check-ups in 2014 at baseline and 2019 at follow-up. We first excluded 1479 participants without oral examinations in baseline or internal medicine examination, and then we excluded 480 participants with history of CHD, stroke, cancer or rheumatoid arthritis in baseline. The remaining 4591 participants were enrolled in final analysis. The flowchart of the study is summarized in Fig. 1.

### Data collection and definitions

Questionnaire interviews and anthropometric and laboratory measurements were performed at baseline and follow-up with the consent of all participants. The demographic characteristics and lifestyle information were collected via a standard questionnaire by our trained staff, including age, sex, education, smoking and drinking status. Smoking and drinking status were defined as ‘current’ and ‘never or former’. Education was defined as ‘below high school’ and ‘high school or above’. Physical activity was classified as ‘Moderate or higher’ (> 80 min per weak) and ‘None or mild’ (< 80 min per week or none).

History of diabetes, hypertension, and periodontitis, the physical and biochemical examination data at baseline collected from the electronic medical record system. Periodontitis cases were defined as having a probing pocket depth greater than 3 mm, with probing bleeding, clinical attachment loss, and absorption of alveolar bone. Diabetes was defined as fasting serum glucose level ≥ 7.0 mmol/L, random serum glucose level ≥ 11.1 mmol/L, or use of antidiabetic medication. Hypertension was defined as a resting blood pressure exceeding 140/90 mmHg or the use of blood pressure lowering medication. Incident cases of CHD were defined as either (1) myocardial infarction or (2) angina pectoris, or (3) silent myocardial ischemia, or (4) ischemic cardiomyopathy in the follow-up medical record. All examinations were performed by physicians.

Body mass index (BMI) was calculated as weight (in kilograms)/height^2 (in metres squared). Blood samples were collected from participants after an overnight fast of at least 12 h. Fasting laboratory measurements included uric acid (UA), total cholesterol (TC), triglycerides (TG), low-density lipoprotein (LDL-c), high-density lipoprotein (HDL-c), creatinine (CREA), glutamic-pyruvic transaminase (ALT), glutamic-oxalacetic transaminase (AST), globulin (GLB), C-reactive protein (CRP), hemoglobin (HGB), and total protein (TP). Blood samples were measured by enzymatic method using a chemistry analyzer (Beckman LX 20, America) at the central laboratory of the hospital.

### Statistical analyses

Data were presented as mean (standard deviation) for continuous variables. Categorical variable was described as number. We used the Wilcoxon signed-rank test (for continuous variables), or the Chi-squared test (for categorical variables) to investigate differences in characteristics at baseline between participants with periodontitis and without periodontitis.

The association between CHD and periodontitis was estimated using relative risk (RR) calculated using modified Poisson regression [12]. All potential confounding variables in the current regression analyses were collected at baseline. Model 1 was adjusted for age and sex. Then, Model 2 was adjusted for age, sex, BMI and history of diabetes. Model 3 was adjusted for UA, TG, TC, CREA, GLB, TP, ALT, AST, and HGB additionally. Lastly, based on Model3, Model 4 was adjusted for education, smoking, drinking, and physical activity. To reduce potential bias caused by including only participants with complete information and exploit the information in incomplete record participants, we used the multiple imputation implemented in the R package Mice [13] to get robust estimates. Missing values are provided in Table S1 (Additional file 1).

We used multiple mediation analysis implanted by the mma package [14] to differentiate the relative effects (RE) from different risk factors on the effect of periodontitis on CHD. Mediation analysis refers to the statistical techniques attempting to make inferences on mediation/confounding effects (effects from X to Y through different paths) [15]. Direct effect of periodontitis is interpreted as the remaining outcome disparity if distributions of various risk factors across periodontitis and non-periodontitis groups could be equalized. The indirect effect (IE) from a certain risk factor (mediator/confounder) is the change in the outcome disparity if the distributions of the risk factor can be set as the same across periodontitis and non-periodontitis groups, while distributions for other risk factors are kept as observed. RE is defined as the ratio of the indirect or direct effect over the total effect. We used the multivariate additive regression trees (MART) to fit variable relationships.

The mma package also provides generic functions to help identify the mediators/confounders and covariate. It tested the significance of two associations: (1) between periodontitis and the potential mediator/confounder; and (2) between the potential mediator/confounder and CHD, when other variables are controlled. For this selection process, we set the significance level at 0.25 to reduce the risk of falsely ignoring important variables. The confidence intervals were calculated based on 200 bootstrap samples. All analyses were performed using R Studio Version 1.1.423. *p* < 0.05(2-sided) was considered statistically significant.

## Results

The final analysis included 4591 individuals. Average age at baseline was 53.9 years. During the follow-up period, 133 participants were diagnosed with CHD. At baseline, 1268 (27.6%) participants were diagnosed with periodontitis. During the follow-up period, 55 patients developed CHD from among those with periodontitis. In the non-periodontitis group, CHD occurred in 78 patients. A significant association was seen between periodontitis at baseline and incident CHD (*p* < 0.001). The detailed information of the baseline characteristics was presented in Table 1.

**Table 1:** Baseline characteristics of the study population

Variable	Total (N = 4591)	Without periodontitis (N = 3323)	With Periodontitis (N = 1268)	*p* Value*
Age(year)	53.9(11)	52.8(10.8)	56.9(10.9)	< 0.001
*Sex*				
Men	3146	2125	1021	< 0.001
Women	1445	1198	247	
BMI	25.5(3.2)	25.4(3.2)	25.8(3.2)	< 0.001
*Education level*				
Below high school	248	163	85	0.024
High school or above	2133	1552	581	
*Current smoking*				
Yes	752	522	230	0.033
No	1506	1111	395	
*Current drinking*				
Yes	1285	909	376	0.09
No	901	668	233	
*Physical activity*				
None or mild	1048	760	288	0.98
Moderate or higher	1134	824	310	
*Hypertension*				
Yes	1816	1249	567	< 0.001
No	2775	2074	701	
*Diabetes*				
Yes	426	260	166	< 0.001
No	4165	3063	1102	
TG (mmol/L)	1.6(1.3)	1.6(1.3)	1.7(1.4)	< 0.001
TC (mmol/L)	4.8(0.9)	4.8(0.9)	4.8(0.9)	0.45
HDL-c (mmol/L)	1.3(0.3)	1.3(0.4)	1.3(0.3)	< 0.001
LDL-c (mmol/L)	3.1(0.8)	3.1(0.8)	3.1(0.8)	0.90
UA (µmol/L)	344.1(85.8)	340.7(86.4)	353.0(83.6)	< 0.001
GLB (g/L)	26.4(3.4)	26.3(3.4)	26.5(3.3)	0.051
CREA (μmol/L)	75.8(16.1)	74.8(15.3)	78.3 (17.9)	< 0.001
ALT (U/L)	21.1(12.2)	21.1(12.2)	21.2(12.2)	0.18
AST (U/L)	20.1(7.0)	20.1(7.0)	20.2(7.1)	0.63
CRP (mg/L)	1.3(2.7)	1.3(2.9)	1.3(2.0)	0.17
HGB (g/L)	150.7(15.4)	149.6(15.7)	153.5(14.1)	< 0.001
TP (g/L)	72.7(3.9)	72.6(3.9)	72.8(3.9)	0.34

Numerical variables were expressed as mean (SD); categorical variables were expressed as number

Abbreviations: *BMI* body mass index, *UA* uric acid, *TC* total cholesterol, *TG* triglycerides, *LDL-c* low-density lipoprotein, *HDL-c* high-density lipoprotein, *CREA* creatinine, *ALT* glutamic-pyruvic transaminase, *AST* glutamic-oxalacetic transaminase, *GLB* globulin, *CRP* C-reactive protein, *HGB* hemoglobin, *TP* total protein

*Wilcoxon signed-rank test (for continuous variables), or the Chi-squared test (for categorical variables)

The adjusted RRs and 95% CIs of periodontitis for the risk of CHD are shown in Table 2. Periodontitis was weakly associated with the risk of CHD when adjusted for age and sex (RR 1.35; 95% CI 0.95–1.91). In Model 2 and Model 3, periodontitis was weakly associated with an increased risk of CHD before multiple imputation in the participants with incomplete data, although the association were not statistically significant. In the analysis using the imputed dataset and fully adjusted model, we observed that periodontitis was weakly associated with the risk of CHD overall (*p* = 0.07). Participants with periodontitis at baseline had 37% increased risk of CHD overall compared to those without periodontitis at baseline (RR 1.37; 95% CI 0.96–1.95).

**Table 2:** Results of modified passion regression model for periodontitis and CHD with their relative risks (RRs) and 95% confidence intervals (CIs)

	N*	Model with original data		Model with imputed data	
		RR (95%CI)	*p* Value	RR (95%CI)	*p* Value
Model 1	4591	1.35(0.95,1.91)	0.08	–	–
Model 2	4357	1.34(0.93,1.90)	0.10	1.31(0.92,1.85)	0.12
Model 3	3208	1.34(0.89,2.00)	0.15	1.32(0.93,1.86)	0.11
Model 4	1651	1.19(0.72,1.97)	0.49	1.37(0.96,1.95)	0.07

Model 1 adjusted for age and sex

Model 2 model 1 and BMI and history of diabetes

Model 3 model 2 and uric acid (UA), total cholesterol (TC), triglycerides (TG), creatinine (CREA), glutamic-pyruvic transaminase (ALT), glutamic-oxalacetic transaminase (AST), globulin (GLB), hemoglobin (HGB), and total protein (TP)

Model 4 model 3 and education, smoking, drinking, and physical activity

*The number of participants with the complete information for models

**Fig. 2:**
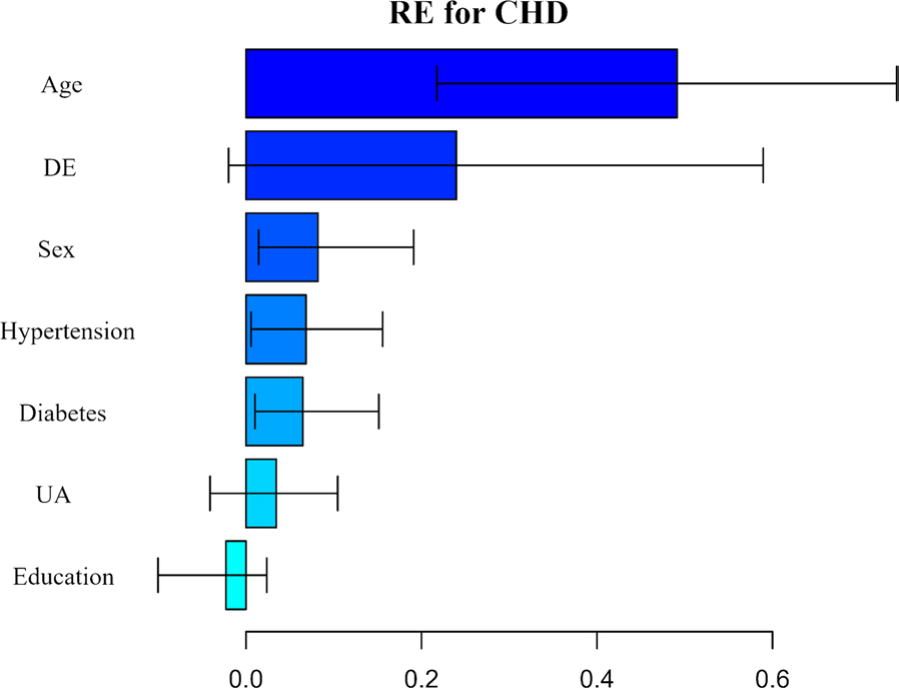
Estimated relative effects (RE) on the effect of periodontitis on CHD from the multiple mediation analysis. RE is defined as the ratio of the indirect effect of different risk factors on the total effect of periodontitis on CHD. DE represents the ratio of the direct effect of periodontitis on the total effect of periodontitis on CHD. Abbreviations: *DE* direct effect, *UA* uric acid

The test results and identified potential mediators/confounders was shown in Table S2 (Additional file 1). Age, sex, history of diabetes, history of hypertension, UA, and education were chosen as potential mediator/confounder. Figure 2 shows the RE for the CHD from the MART model. If the “Age” could be set equivalent among participants with and without periodontitis, the effect of periodontitis on CHD would reduce by 49%. Other variables such as sex (8%), history of diabetes (6%), and history of hypertension (6%) also significantly explain the association. An interesting variable is education, which have a negative relative effect (− 2%) (opposite to the total effect), but this association were not statistically significant (95% CI − 0.10 to 0.02). All the mediators/confounders explained most of the effect of periodontitis on CHD (RE 0.76; 95% CI 0.41–1.02). The detailed results of the multiple mediation analysis were presented in Table S3 (Additional file 1).

**Fig. 3:**
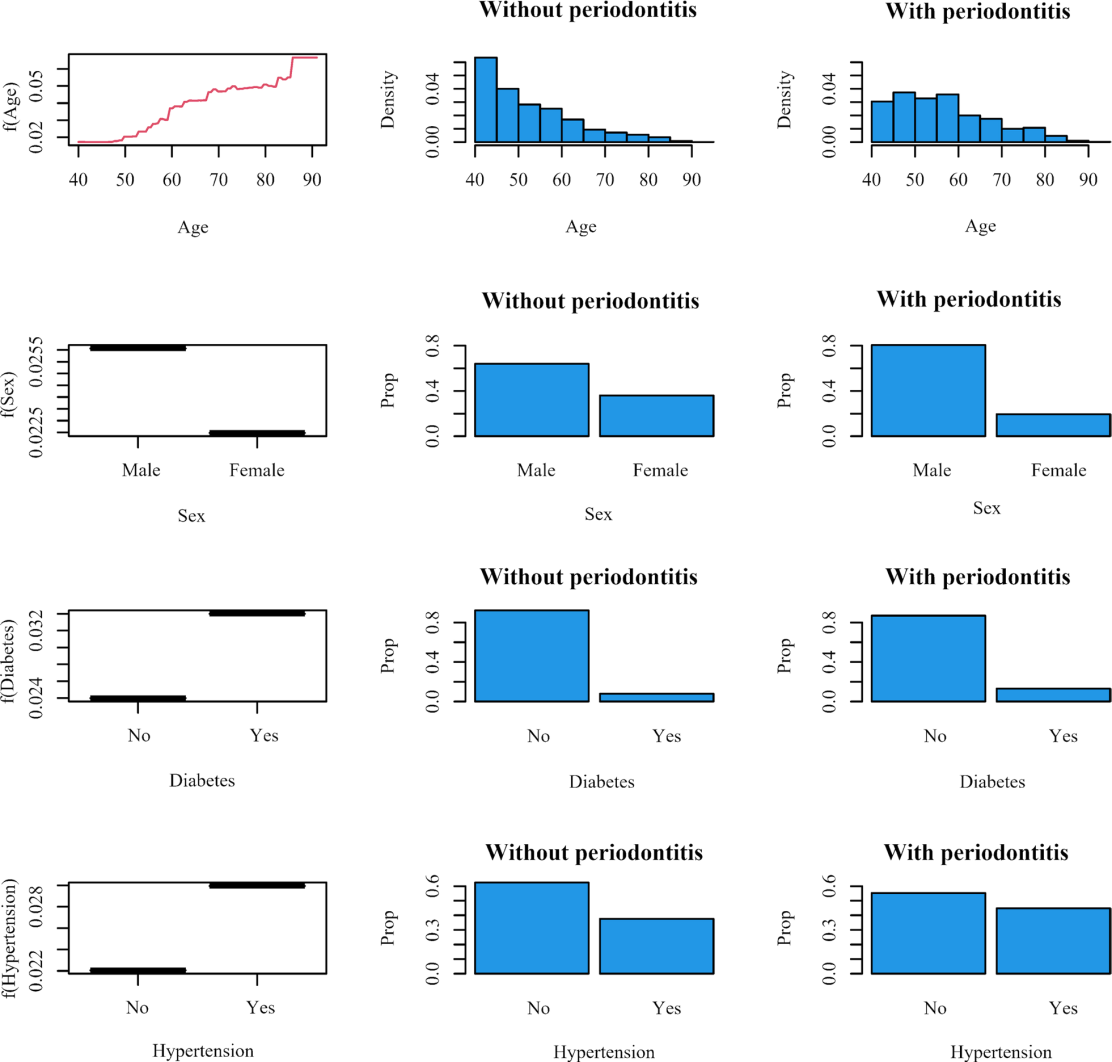
The marginal effect of the age, sex, history of diabetes and history of hypertension in additive regression trees (MART) model, and the distribution of the variables in participants with and without periodontitis at baseline

Figure 3 shows the marginal effect of the significant variables in MART model, and the distribution of the variables in participants with and without periodontitis at baseline, respectively. Compared with those without periodontitis at baseline, participants with periodontitis at baseline have more older participants, male, and higher prevalence of diabetes and hypertension. All those factors were associated with an increased risk of CHD.

## Discussion

The main finding of this prospective cohort study was that periodontitis was weakly associated with an increased risk for CHD among the middled-aged and elderly in China. Previous epidemic studies in other regions have shown associations between periodontitis and CHD, and most of existing ones are biased towards periodontitis is a risk factor for CHD [5]. However, some studies found no significant relationship between periodontitis and CHD [16–18]. This may be attributed to differences in the target population and the definition of periodontitis. In some studies, periodontitis was self-reported, and then no significant results were found [16, 18]. Some studies found significant results when periodontal pocket was used as a main indicator of periodontitis [19, 20]. Basing the Centers for Disease Control and Prevention in partnership with the American Academy of Periodontology case definitions [21], Niramol et al. found a significant association between severe periodontitis and the incidence of CHD [22]. In our study, periodontal pocket depth greater than 3 mm is a main indicator for periodontitis.

We noted that a stronger association was obtained when the missing data in Model 4 were simulated. However, the result obtained from original data was not significant. The likely reason for this is that the participants who completed questionnaires had a degree of heterogeneity. In these people, 2133 participants had received the high school or above education, and only 248 participants had received the below high school education. When we fitted model adjusted only for age and sex using this data, the result was not significant (RR 1.25; 95% CI 0.78–1.99). After applying multiple imputation, potential bias caused by including only participants with complete information were minimized.

We observed that age, sex, history of diabetes, and history of hypertension have a significant indirect effect in explaining the effect of periodontitis on CHD. Almost half of the effect of periodontitis on CHD that can be explained by age. It should be noted that the age is reported in years, which means age may explain more disparity for this association. For sex, previous studies identified that men disproportionately develop periodontal diseases due to a combination of biological and gender related reasons including immune system factors, hormone differences, poorer oral hygiene behaviors, and greater tobacco use [23]. Compared with women, men also reported a significantly higher prevalence of CHD [24, 25]. For diabetes, some epidemiological studies and reviews have reported that periodontitis is a potential risk factor for diabetes mellitus. In fact, early blood glucose fluctuations are thought to be associated with development of poor oral health [26]; There may be a bidirectional association between oral health and type 2 diabetes [27]. Meanwhile, most patients who have type 2 diabetes mellitus develop vascular complications [28]. For hypertension, the occurrence of periodontitis leads to an increase in blood pressure [29]. Periodontitis can also lead to ineffectiveness of antihypertensive [30, 31]. Hypertension is also a risk factor for CHD among middle to old age [32, 33].

In addition, we also found UA is a potential mediator. Epidemiology studies suggested that UA levels were positively associated with periodontitis [34, 35]. Porphyromonas gingivalis is a major periodontopathogen, and its gingipain proteases play a critical role in the pathogenesis of periodontitis. gingipain-induced UA can mediate inflammation in periodontal tissue cells [36]. UA is also associated with the risk of incident CHD [37]. The role of UA in the link between periodontitis and CHD requires further study.

To the best of our knowledge, this is the first cohort study investigation of the association between periodontitis and CHD among the middled-aged and elderly in China, and we first used multiple mediation analysis to quantify the relative effects from different risk factors on the effect of periodontitis on CHD. This study will enhance our understanding of the association between CHD and periodontitis, and provide epidemiologic evidence in Chinese population. However, this study has some limitations. First, oxidative stress [38] and genetic factors [39, 40] may also mediate the association. We did not collect relevant variables. Second, we did not distinguish the severity of periodontitis. Third, among those who completed the questionnaire, better-educated people were vastly outnumbered by Less educated people. Moreover, the observed associations of this single-center study needed further validation in other cohorts.

## Conclusion

In summary, periodontitis was weakly associated with an increased risk of CHD among the middled-aged and elderly in China. However, most of the association can be explained by age, sex, history of diabetes, history of hypertension, UA and education. Further studies are required to identify more mediators and elucidate the mechanisms of how periodontitis increases the risk of CHD.

## Supplementary Information

**Additional file 1**: Summary of missing values, potential mediators/confounders and covariates, summary of mediation/confounding effect estimations for periodontitis in CHD, and baseline characteristics of the study population categorized by CHD event.

## Data Availability

The datasets generated during and/or analyzed during the current study are available from the corresponding author on reasonable request.

## References

[1] Kinane DF, Stathopoulou PG, Papapanou PN (2017). Periodontal diseases. Nat Rev Dis Primers.

[2] Cardoso EM, Reis C, Manzanares-Céspedes MC (2018). Chronic periodontitis, inflammatory cytokines, and interrelationship with other chronic diseases. Postgrad Med.

[3] Kiechl S, Egger G, Mayr M, Wiedermann CJ, Bonora E, Oberhollenzer F, Muggeo M, Xu Q, Wick G, Poewe W (2001). Chronic infections and the risk of carotid atherosclerosis: prospective results from a large population study. Circulation.

[4] Beck JD, Philips K, Moss K, Sen S, Morelli T, Preisser J, Pankow J (2020). Periodontal disease classifications and incident coronary heart disease in the Atherosclerosis Risk in Communities study. J Periodontol.

[5] Gao S, Tian J, Li Y, Liu T, Li R, Yang L, Xing Z (2021). Periodontitis and number of teeth in the risk of coronary heart disease: an updated meta-analysis. Med Sci Monit.

[6] Winning L, Patterson CC, Linden K, Evans A, Yarnel J, McKeown PP, Kee F, Linden GJ (2020). Periodontitis and risk of prevalent and incident coronary heart disease events. J Clin Periodontol.

[7] Dietrich T, Sharma P, Walter C, Weston P, Beck J (2013). The epidemiological evidence behind the association between periodontitis and incident atherosclerotic cardiovascular disease. J Periodontol.

[8] Lockhart PB, Bolger AF, Papapanou PN, Osinbowale O, Trevisan M, Levison ME, Taubert KA, Newburger JW, Gornik HL, Gewitz MH (2012). Periodontal disease and atherosclerotic vascular disease: does the evidence support an independent association? A scientific statement from the American Heart Association. Circulation.

[9] Zhou X, Xu X, Li J, Hu D, Hu T, Yin W, Fan Y, Zhang X (2018). Oral health in China: from vision to action. Int J Oral Sci.

[10] Zhang X, Lu Z, Liu L (2008). Coronary heart disease in China. Heart.

[11] Liu J, Zhao Z, Mu Y, Zou X, Zou D, Zhang J, Chen S, Tao L, Guo X (2018). Gender differences in the association between serum uric acid and prediabetes: a six-year longitudinal cohort study. Int J Environ Res Public Health.

[12] Zou G (2004). A modified poisson regression approach to prospective studies with binary data. Am J Epidemiol.

[13] Buuren SV. Groothuis-Oudshoorn K: MICE: multivariate imputation by chained equations in R. J Stat Softw 2011;45(3).

[14] Yu Q, Li B. mma: An R package for mediation analysis with multiple mediators. J Open Res Softw 2017;5(2).

[15] Yu Q, Medeiros KL, Wu X, Jensen RE (2018). Nonlinear predictive models for multiple mediation analysis: With an application to explore ethnic disparities in anxiety and depression among cancer survivors. Psychometrika.

[16] Howell TH, Ridker PM, Ajani UA, Hennekens CH, Christen WG (2001). Periodontal disease and risk of subsequent cardiovascular disease in U.S. male physicians. J Am Coll Cardiol.

[17] Hujoel PP, Drangsholt M, Spiekerman C, DeRouen TA (2000). Periodontal disease and coronary heart disease risk. Jama.

[18] Noguchi S, Toyokawa S, Miyoshi Y, Suyama Y, Inoue K, Kobayashi Y (2015). Five-year follow-up study of the association between periodontal disease and myocardial infarction among Japanese male workers: MY Health Up Study. J Public Health (Oxf).

[19] DeStefano F, Anda RF, Kahn HS, Williamson DF, Russell CM (1993). Dental disease and risk of coronary heart disease and mortality. BMJ.

[20] Morrison HI, Taylor GW (1999). Periodontal disease and risk of fatal coronary heart and cerebrovascular diseases. J Cardiovasc Risk.

[21] Eke PI, Page RC, Wei L, Thornton-Evans G, Genco RJ (2012). Update of the case definitions for population-based surveillance of periodontitis. J Periodontol.

[22] Tiensripojamarn N, Lertpimonchai A, Tavedhikul K, Udomsak A, Vathesatogkit P, Sritara P, Charatkulangkun O (2021). Periodontitis is associated with cardiovascular diseases: a 13-year study. J Clin Periodontol.

[23] Lipsky MS, Su S, Crespo CJ, Hung M (2021). Men and oral health: a review of sex and gender differences. Am J Mens Health.

[24] Jousilahti P, Vartiainen E, Tuomilehto J, Puska P (1999). Sex, age, cardiovascular risk factors, and coronary heart disease: a prospective follow-up study of 14 786 middle-aged men and women in Finland. Circulation.

[25] Lernfelt B, Landahl S, Svanborg A (1990). Coronary heart disease at 70, 75 and 79 years of age: a longitudinal study with special reference to sex differences and mortality. Age Ageing.

[26] Verhulst MJ, Loos BG, Gerdes VE, Teeuw WJ (2019). Evaluating all potential oral complications of diabetes mellitus. Front Endocrinol.

[27] Beck J, Papapanou P, Philips K, Offenbacher S (2019). Periodontal medicine: 100 years of progress. Journal of dental research.

[28] Stolar M (2010). Glycemic control and complications in type 2 diabetes mellitus. American journal of medicine.

[29] Munoz Aguilera E, Suvan J, Buti J, Czesnikiewicz-Guzik M, Barbosa Ribeiro A, Orlandi M, Guzik TJ, Hingorani AD, Nart J, D'Aiuto F (2020). Periodontitis is associated with hypertension: a systematic review and meta-analysis. Cardiovasc Res.

[30] Surma S, Romanczyk M, Witalinska-Labuzek J, Czerniuk MR, Labuzek K, Filipiak KJ (2021). Periodontitis, blood pressure, and the risk and control of arterial hypertension: epidemiological, clinical, and pathophysiological aspects-review of the literature and clinical trials. Curr Hypertens Rep.

[31] Munoz Aguilera E, Suvan J, Orlandi M, Miro Catalina Q, Nart J, D'Aiuto F (2021). Association between periodontitis and blood pressure highlighted in systemically healthy individuals: results from a nested case-control study. Hypertension.

[32] Liu M, Zhang S, Chen X, Zhong X, Xiong Z, Yang D, Lin Y, Huang Y, Li Y, Wang L (2021). Association of mid- to late-life blood pressure patterns with risk of subsequent coronary heart disease and death. Front Cardiovasc Med.

[33] Zhang Y, Jiang X, Bo J, Yin L, Chen H, Wang Y, Yu H, Wang X, Li W (2018). Investigators PU-C: Risk of stroke and coronary heart disease among various levels of blood pressure in diabetic and nondiabetic Chinese patients. J Hypertens.

[34] Banu S, Jabir NR, Mohan R, Manjunath NC, Kamal MA, Kumar KR, Zaidi SK, Khan MS, Tabrez S (2015). Correlation of Toll-like receptor 4, interleukin-18, transaminases, and uric acid in patients with chronic periodontitis and healthy adults. J Periodontol.

[35] Byun SH, Yoo DM, Lee JW, Choi HG. Analyzing the Association between Hyperuricemia and Periodontitis: a cross-sectional study using KoGES HEXA data. Int J Environ Res Public Health 2020;17(13). 10.3390/ijerph17134777 PMC7370102 32630802

[36] Jun HK, An SJ, Kim HY, Choi BK (2020). Inflammatory response of uric acid produced by Porphyromonas gingivalis gingipains. Mol Oral Microbiol&nbsp;.

[37] Ndrepepa G (2018). Uric acid and cardiovascular disease. Clinica Chimica Acta.

[38] Wang Y, Andrukhov O, Rausch-Fan X (2017). Oxidative stress and antioxidant system in periodontitis. &nbsp;Front Physiol.

[39] Schaefer AS, Richter GM, Groessner-Schreiber B, Noack B, Nothnagel M, El Mokhtari NE, Loos BG, Jepsen S, Schreiber S (2009). Identification of a shared genetic susceptibility locus for coronary heart disease and periodontitis. PLoS Genet.

[40] Mucci LA, Hsieh CC, Williams PL, Arora M, Adami HO, de Faire U, Douglass CW, Pedersen NL (2009). Do genetic factors explain the association between poor oral health and cardiovascular disease? A prospective study among Swedish twins. Am J Epidemiol.

