# Supplementary material for: Risk of coronary heart disease in patients with periodontitis among the middled-aged and elderly in China: a cohort study
Source: BMC Oral Health. 2021 Dec 7;21:621. doi: 10.1186/s12903-021-01951-z (PMC8650264; doi:10.1186/s12903-021-01951-z)
Supplement: Supplementary file 1 — Additional file 1: Summary of missing values, potential mediators/confounders and covariates, summary of mediation/confounding effect estimations for periodontitis in CHD, and baseline characteristics of the study population categorized by CHD event. [file 12903_2021_1951_MOESM1_ESM.docx]

Table S1. Summary of missing values.

| Variable | Missing values(N) |
| --- | --- |
| BMI | 234 |
| Education | 2,210 |
| Current smoking | 2,333 |
| Current drinking | 2,405 |
| Physical activity | 2,409 |
| TG | 17 |
| TC | 17 |
| HDL-c | 17 |
| LDL-c | 17 |
| UA | 17 |
| GLB | 1,193 |
| CREA | 17 |
| ALT | 17 |
| AST | 1,193 |
| CRP | 2,673 |
| HGB | 21 |
| TP | 1,193 |

Abbreviations: BMI, body mass index; UA, uric acid; TC, total cholesterol; TG, triglycerides; LDL-c, low-density lipoprotein; HDL-c, high-density lipoprotein; CREA, creatinine; ALT, glutamic-pyruvic transaminase, AST, glutamic-oxalacetic transaminase; GLB, globulin; CRP, C-reactive protein; HGB, hemoglobin; TP, total protein.

Table S2. Potential mediators/confounders.

| Variable | *p*-value | *p*-value |
| --- | --- | --- |
| Age* | 0.052 | <0.001 |
| Sex* | 0.056 | <0.001 |
| Education* | 0.157 | 0.02 |
| Diabetes* | 0.042 | <0.001 |
| Hypertension* | 0.163 | <0.001 |
| Smoking | 0.852 | - |
| Drinking | 0.278 | - |
| Physical activity | 0.918 | - |
| BMI | 0.808 | - |
| TG | 0.283 | - |
| HDL-c | 0.657 | - |
| LDL-c | 0.343 | - |
| TC | 0.437 | - |
| HGB | 0.731 | - |
| UA* | 0.042 | <0.001 |
| ALT | 0.832 | - |
| CREA | 0.582 | - |
| GLB | 0.498 | - |
| TP | 0.674 | - |
| AST**^−^** | 0.303 | - |
| CRP | 0.829 | - |

Columns 2 show the *p* values of Type III tests for the corresponding variables in the full model in predicting coronary heart disease (CHD). Column 3 shows the *p* value for testing the association between periodontitis and the corresponding variable.

Abbreviations: BMI, body mass index; UA, uric acid; TC, total cholesterol; TG, triglycerides; LDL-c, low-density lipoprotein; HDL-c, high-density lipoprotein; CREA, creatinine; ALT, glutamic-pyruvic transaminase, AST, glutamic-oxalacetic transaminase; GLB, globulin; CRP, C-reactive protein; HGB, hemoglobin; TP, total protein.

* The variable is identified as a potential mediator/confounder.

Table S3. Summary of mediation/confounding effect estimations for periodontitis in CHD**.**

| Mediator/confounder | IE (95%CI) | RE (95%CI) |
| --- | --- | --- |
| Age | 0.16(0.10,0.24) | 0.49(0.21,0.74) |
| Sex | 0.02(0.01,0.06) | 0.08(0.01, 0.19) |
| Diabetes | 0.02(0.01,0.04) | 0.06(0.01,0.15) |
| Hypertension | 0.02(0.01,0.05) | 0.06(0.01,0.15) |
| Education | -0.01(-0.03,0.01) | -0.02(-0.10,0.02) |
| UA | 0.01(-0.01,0.03) | 0.03(-0.04,0.10) |
| Indirect effect | 0.26(0.19,0.36) | 0.76(0.41,1.02) |
| Direct effect | - | 0.24(-0.02,0.58) |

Abbreviations: IE, indirect effect; RE, relative effect; UA, uric acid.

RE is defined as the ratio of the indirect or direct effect over the total effect.

Table S4. Baseline characteristics of the study population, categorized by CHD event.

| Variable | Total  (N = 4,591) | No incident CHD  (N = 4,458) | Incident CHD  (N = 133) | *p*-value^*^ |
| --- | --- | --- | --- | --- |
| **Age(year)** | 53.9(11) | 53.6(10.9) | 62.6(11.2) | <0.001 |
| **Sex** |  |  |  |  |
| Men | 3,146 | 3,031 | 115 | <0.001 |
| Women | 1,445 | 1,427 | 18 |  |
| **BMI** | 25.5(3.2) | 25.5(3.2) | 26.5(3.6) | 0.003 |
| **Education level** |  |  |  |  |
| Below high school | 248 | 243 | 5 | 0.36 |
| High school or above | 2,133 | 2,062 | 71 |  |
| **Current smoking** |  |  |  |  |
| Yes | 752 | 733 | 19 | 0.26 |
| No | 1,506 | 1,453 | 53 |  |
| **Current drinking** |  |  |  |  |
| Yes | 1,285 | 1,247 | 38 | 0.18 |
| No | 901 | 864 | 37 |  |
| **Physical activity** |  |  |  |  |
| None or mild | 1,048 | 1,009 | 39 | 0.15 |
| Moderate or higher | 1,134 | 1,105 | 29 |  |
| **Periodontitis** |  |  |  |  |
| Yes | 1,268 | 1,213 | 55 | <0.001 |
| No | 3,323 | 3,245 | 78 |  |
| **Hypertension** |  |  |  |  |
| Yes | 1,816 | 1,724 | 92 | <0.001 |
| No | 2,775 | 2,734 | 41 |  |
| **Diabetes** |  |  |  |  |
| Yes | 426 | 392 | 34 | <0.001 |
| No | 4,165 | 4,066 | 99 |  |
| **TG (mmol/L)** | 1.6(1.3) | 1.6(1.3) | 1.8(1.2) | 0.049 |
| **TC (mmol/L)** | 4.8(0.9) | 4.8(0.9) | 4.8(1.1) | 0.28 |
| **HDL-c (mmol/L)** | 1.3(0.3) | 1.3(0.3) | 1.2(0.3) | 0.002 |
| **LDL-c (mmol/L)** | 3.1(0.8) | 3.1(0.8) | 3.0(1.0) | 0.24 |
| **UA (µmol/L)** | 344.1(85.8) | 343.3(85.9) | 370.6(78.0) | <0.001 |
| **GLB (g/L)** | 26.4(3.4) | 26.4(3.4) | 26.2(3.5) | 0.40 |
| **CREA (μmol/L)** | 75.8(16.1) | 75.7(16.2) | 79.4(14.8) | 0.008 |
| **ALT (U/L)** | 21.1(12.2) | 21.0(12.0) | 23.3(17.3) | 0.074 |
| **AST (U/L)** | 20.1(7.0) | 20.1(6.9) | 21.9(10.3) | 0.016 |
| **CRP (mg/L)** | 1.3(2.7) | 1.3(2.6) | 1.8(4.6) | 0.26 |
| **HGB (g/L)** | 150.7(15.4) | 150.6(15.4) | 153.3(13.2) | 0.043 |
| **TP (g/L)** | 72.7(3.9) | 72.7(3.9) | 72.5(4.1) | 0.42 |

Numerical variables were expressed as mean (SD); categorical variables were expressed as number.

Abbreviations: BMI, body mass index; UA, uric acid; TC, total cholesterol; TG, triglycerides; LDL-c, low-density lipoprotein; HDL-c, high-density lipoprotein; CREA, creatinine; ALT, glutamic-pyruvic transaminase, AST, glutamic-oxalacetic transaminase; GLB, globulin; CRP, C-reactive protein; HGB, hemoglobin; TP, total protein.

*Wilcoxon signed-rank test (for continuous variables), or the Chi-squared test (for categorical variables).
